# De novo assembly and Characterisation of the Transcriptome during seed development, and generation of genic-SSR markers in Peanut (*Arachis hypogaea *L.)

**DOI:** 10.1186/1471-2164-13-90

**Published:** 2012-03-12

**Authors:** Jianan Zhang, Shan Liang, Jialei Duan, Jin Wang, Silong Chen, Zengshu Cheng, Qiang Zhang, Xuanqiang Liang, Yurong Li

**Affiliations:** 1 Institute of Food and Oil Crops, Hebei Academy of Agriculture and Forestry Sciences/Laboratory of Crop Genetics and Breeding of Hebei Province, Shijiazhuang 050031, China; 2 National Millet Improvement Center of China, Institute of Millet Crops, Hebei Academy of Agriculture and Forestry Sciences, Shijiazhuang 050031, China; 3 Protein Science Laboratory of the Ministry of Education, School of Life Sciences, Tsinghua University, Beijing 100084, China; 4 Key Laboratory of Crop Germplasm Resources and Utilization, Ministry of Agriculture/The National Key Facility for Crop Gene Resources and Genetic Improvement/Institute of Crop Science, Chinese Academy of Agricultural Sciences, Beijing 100081, China; 5 Crops Research Institute, Guangdong Academy of Agricultural Sciences, Guangdong 510000, China

## Abstract

**Background:**

The peanut (*Arachis hypogaea *L.) is an important oilseed crop in tropical and subtropical regions of the world. However, little about the molecular biology of the peanut is currently known. Recently, next-generation sequencing technology, termed RNA-seq, has provided a powerful approach for analysing the transcriptome, and for shedding light on the molecular biology of peanut.

**Results:**

In this study, we employed RNA-seq to analyse the transcriptomes of the immature seeds of three different peanut varieties with different oil contents. A total of 26.1-27.2 million paired-end reads with lengths of 100 bp were generated from the three varieties and 59,077 unigenes were assembled with N50 of 823 bp. Based on sequence similarity search with known proteins, a total of 40,100 genes were identified. Among these unigenes, only 8,252 unigenes were annotated with 42 gene ontology (GO) functional categories. And 18,028 unigenes mapped to 125 pathways by searching against the Kyoto Encyclopedia of Genes and Genomes pathway database (KEGG). In addition, 3,919 microsatellite markers were developed in the unigene library, and 160 PCR primers of SSR loci were used for validation of the amplification and the polymorphism.

**Conclusion:**

We completed a successful global analysis of the peanut transcriptome using RNA-seq, a large number of unigenes were assembled, and almost four thousand SSR primers were developed. These data will facilitate gene discovery and functional genomic studies of the peanut plant. In addition, this study provides insight into the complex transcriptome of the peanut and established a biotechnological platform for future research.

## Background

The peanut (*Arachis hypogaea *L.), also known as the groundnut, is an important oilseed crop in the tropical and subtropical regions of the world. It is grown on six continents but mainly in Asia, Africa and America. Peanuts are cultivated on 23.51 million hectares worldwide, with a total global production of approximately 35.52 million tons (the weight includes the shell). China is the largest producer in the world, accounting for 37.6% (13.34 million tons) of the total world production (FAO, 2009, http://faostat.fao.org).

Peanuts have a desirable fatty acid profile and are rich in vitamins, minerals and bioactive materials, including several known heart-healthy nutrients, such as monounsaturated and polyunsaturated fatty acids, potassium, magnesium, copper niacin, arginine, fibre, α-tocopherol, folates, phytosterols, and flavonoids. Indeed, peanut consumption has been associated with an improvement in the overall quality of the diet and nutrient [1-4].

In China, almost 60% of the peanuts are used to produce peanut oil [5]. Peanut oil, due to its high monounsaturated fat content, is considered healthier than saturated oils and is resistant to rancidity. Monounsaturated fat, much of which is oleic acid, is a healthy type of fat that has been implicated in the health of skin [6] and has been demonstrated to reduce cardiovascular disease risk and/or risk factors in both epidemiological and clinical studies [1,2,7].

The development of the peanut seed has been studied intensely to understand the physiological, biochemical, and molecular characteristics that determine the oil quality and their beneficial nutritional contributions. However, the development of the peanut seed is a complex process involving a cascade of biochemical changes, which involve the transcriptional modulation of many genes, yet little is known about these transcriptional changes and their regulation. To date, little research in this area has been reported. Bi [8] developed a seed cDNA library for the peanut to analyse gene expression levels during seed development, and 17,000 expressed sequence tags (ESTs) were sequenced and used for microarray analysis. Recently, the development of next-generation high-throughput DNA sequencing technology has provided a novel method for both mapping and quantifying transcriptomes (RNA-seq) [9]. RNA-seq technology has been successfully applied quite ubiquitously to species such as humans, yeast, mice, grape, *Arabidopsis*, rice, soybeans, sesame, and sweetpotato [9-19]. Moreover, RNA-seq data are highly reproducible, with few systematic discrepancies among technical replicates [20]. The latest paired-end tag sequencing strategy of RNA-seq further improves the DNA sequencing efficiency and expands short-read lengths, providing a better depiction of transcriptomes [21]. Transcriptomic information is used in a wide range of biological studies and provides fundamental insight into biological processes and applications, such as the levels of gene expression [22], the gene expression profiles during development [13,17] or after experimental treatments [23], gene discovery [24], SSR mining [10,11,25], and SNP discovery [12,25-27]. However, transcriptomic information is lacking for the peanut plant because this information is difficult to obtain and, to date, there has been little interest in such data.

Chen [28] reported that the accumulation of seed oil in peanuts could be divided into three stages based on phenotype, namely, the initial accumulation stage, the fast accumulation stage and the steady accumulation stage. As we are interested in identifying genes that are expressed in the seed during the fast accumulation period, we carried out a global analysis of the peanut transcriptome during seed development using the Illumina RNA-seq method. We also present an overview of the RNA-seq data for the peanut as a potential model for future RNA-seq analyses and to establish a biotechnological platform for peanut research.

## Methods

### Sample Preparation and Sequencing

Three peanut varieties, which greatly differ in oil content but are similar in mature process, were used in this study. Jihua 4 (JH4), a variety with a high oil content (57%), as determined in a 2009 field experiment in Hebei, China, was bred at the Hebei Institute of Agricultural Sciences and is widely grown in Northern China. Kaixuan01-6 (K01), a germplasm resource with a similar oil content (52%), also determined in the above field experiment in Hebei, was provided by the Yantai Institute of Agricultural Sciences in the Shandong Province of China. Te21 (T21), a germplasm resource with a low oil content (48%), as determined in China, was also used.

For each variety, RNA was isolated from ten immature seeds of five plants, which were harvested at 7 weeks after flowering during the 2010 growing season from a farm in Hebei province (Shijiazhuang, Hebei, China). Total RNA was extracted using a previously described method [20]. The RNA quality and quantity were determined using an Agilent 2100 Bioanalyzer (Agilent Technologies, Santa Clara, CA). Beads coated with oligo(dT) were used to isolate poly (A) mRNA after the total RNA was collected. Fragmentation buffer (Ambion, Austin, TX) was added to digest the mRNA to produce short fragments. The first strand of cDNA was synthesised using random hexamer primers, followed by synthesis of the second strand. The short fragments were purified with the QIAquick PCR Purification kit (Qiagen, Valencia, CA) for both end repair and the poly (A) addition reaction. The purified DNA libraries were amplified by PCR for 18 cycles. Finally, Solexa HiSeq™ 2000 was employed to sequence the libraries using PCR amplification (BGI, Shenzhen, China).

### De novo Assembly and Analysis of Illumina Reads

The samples were assembled with SOAPdenovo [29] separately. The numbers of paired-end Illumina reads of JH4, K01, and T21 were 27,159,362, 26,938,530, and 26,142,148, respectively. The reads were first combined to form longer fragments, i.e., contigs. The reads were then mapped back to the contigs, and the paired-end reads and contigs from the same transcript were assembled to form a longer sequence, with N for unknown sequences (i.e., scaffolds). Paired-end reads were again used for gap filling of the scaffolds to obtain unigenes with the least Ns that could not be extended on either end. For future analyses, the unigenes from the three samples were assembled again to acquire non-redundant unigenes (All-Unigenes) that were as long as possible. The All-Unigenes assembled from the three samples were compared with the NCBI non-redundant (NR) protein database using blastx v2.2.14 [30] with an E-value cut-off of 1e^-5^. Based on the results of the protein database annotation, Blast2GO [31] was employed to obtain the functional classification of the unigenes based on GO terms. WEGO software [32] was used to perform the GO functional classification for all of the unigenes and to understand the distribution of the gene functions of this species at the macro level. The KEGG database (V56.0, Oct. 1, 2010) [33] was used to annotate the pathway of these unigenes.

### SSR mining and primer design

We employed MIcroSAtellite (MISA) http://pgrc.ipk-gatersleben.de/misa/ for microsatellite mining. In this study, the SSRs were considered to contain motifs with two to six nucleotides in size and a minimum of 5 contiguous repeat units. Based on MISA results, Primer3 v2.23 (http://primer3.sourceforge.net) was used to design the primer pairs with default setting, and the PCR product size ranging from 100 to 280 bp. Six varieties were selected to validated the polymorphism of 160 random SSR markers. The six varieties included three varieties (Jihua 2, Jihua 5, and SW9721), which were all bred at the Hebei Institute of Agricultural Sciences, and the three varieties (JH4, K01, T21) used in this study. The SSR data on the six varieties were obtained following the methods described by Liang [34].

## Results and Discussion

### Sequencing and De novo Assembly of Solexa Short Reads

We generated 27.2, 26.9 and 26.1 million 100-bp paired-end reads for the JH4, K01 and T21 varieties, encompassing 2.44, 2.42 and 2.35 Gb of sequence data, respectively (Table 1). The GC contents of the three varieties were 48.94%, 49.05%, and 47.94%, respectively.

**Table 1:** Summary of the short reads and the assemblies for three varieties.

Variety	Total Reads	Total Nucleotides (nt)	GC Percentage	Unigenes	Contribute to All-Unigenes
JH4	27,159,362	2,444,342,580	48.94%	44,028	74.53%
K01	26,938,530	2,424,467,700	49.06%	47,110	79.74%
T21	26,142,148	2,352,793,320	47.94%	44,157	74.74%

Assembling these reads produced 44,028, 47,110 and 44,157 unigenes for the JH4, K01 and T21 varieties, respectively; only unigenes greater than 200 bp in length were further analysed. The N50 values of these three unigenes were 664 bp, 616 bp and 616 bp, respectively. After the final clustering, 59,077 unigenes were obtained, with approximately equal contribution from varieties JH4 (74.53%), K01 (79.74%), and T21 (74.74%) (The peanut transcriptome sequences is available at National Centre for Biotechnology Information (NCBI) Transcriptome Shotgun Assembly (TSA) database, http://www.ncbi.nlm.nih.gov/, accession numbers are JR540742-JR590649). The length of the unigenes varied from 200 bp to 10654 bp, with an average of 619 bp, and the N50 value was 823 bp. The majority of the reads were in the range of 201-500 bp (64.80% of the unigenes), and 46,176 unigenes (78.16% of all of the unigenes) were shorter than 1 k. These results should provide a sequence basis for future studies, such as gene cloning and transgenic studies.

### Characterisation of the unigenes

Among the 59,077 unigenes, the sequence directions of 42,997 of the unigenes were determined using blastx against the NCBI non-redundant (NR), Swiss-Prot, KEGG, and clusters of orthologous groups (COG) of protein databases with an E-value cut-off of 1e^-5^. In addition, the protein coding regions of 39,204 unigenes were predicted.

The unigenes were compared against the NCBI NR protein database using blastx. Among the 59,077 unigenes, 40,100 (67.88%) had at least one significant match with an E-value below 1e^-5^. A total of 18,977 unigenes had no significant matches to any known protein, the result that may be partly due to novel genes or highly divergent genes, or these unigenes could represent untranslated regions. For validating redundancy of the data set with publicly available data, sequence similarity search was conducted against the NCBI Unigene database ftp://ftp.ncbi.nlm.nih.gov/repository/UniGene/Arachis_hypogea/ using blastn with an E-value cut-off of 1e^-10^. The results indicated that out of 59,077 unigenes, 34,815 (58.93%) showed significant similarity to publicly unigenes database. All the information on the redundancy of the data set with the publicly available data were showed in the supplemental file (Additional file 1: Table S1).

**Figure 1:**
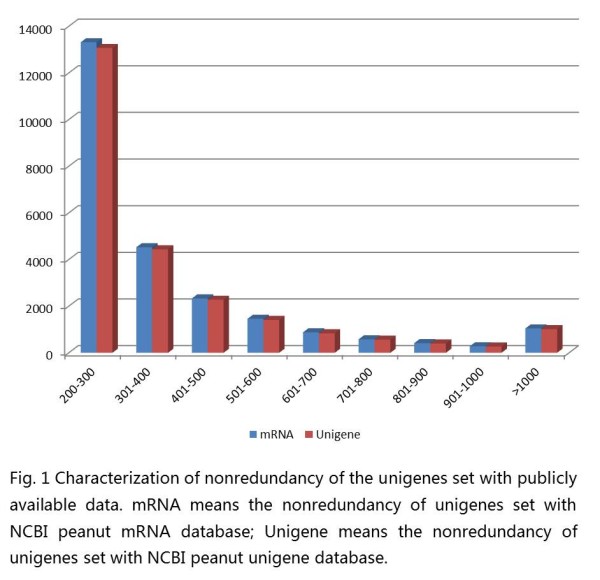
**Characterization of nonredundancy of the unigenes set with publicly available data**. mRNA means the nonredundancy of unigenes set with NCBI peanut mRNA database; Unigene means the nonredundancy of unigenes set with NCBI peanut unigene database.

**Figure 2:**
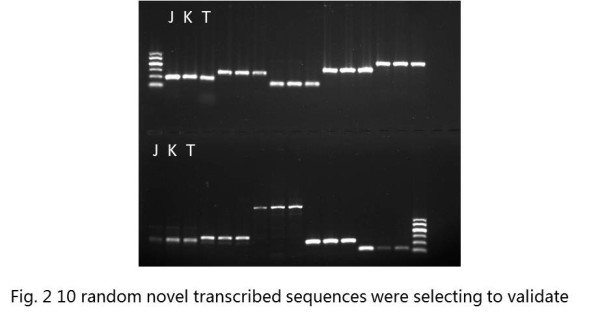
**Validation of novel transcribed sequences**.

We identified novel transcribed sequences using blastn and the NCBI mRNA database sequences for peanut with an E-value cut-off of 1e^-10^. A total of 24,814 (42.00%) of the unigenes did not significantly match the mRNA database and were, thus, considered putative novel transcribed sequences (Figure 1). The lengths of the novel unigenes varied from 200 bp to 6267 bp, with an N50 value of 288 bp. Among these novel unigenes, only 1,046 (4.2%) were longer than 1 kb. A total of 14,407 (58.1% of the novel transcribed sequences) of the unigenes had at least one significant match against the NCBI NR protein database. RT-PCR was carried out to validate these expression distributions further by selecting 10 random novel unigenes (Figure 2). The result indicated that all ten unigenes got right amplifications. The new information provided by this study could be useful to further peanut research.

### Functional classification of the peanut unigenes

**Figure 3:**
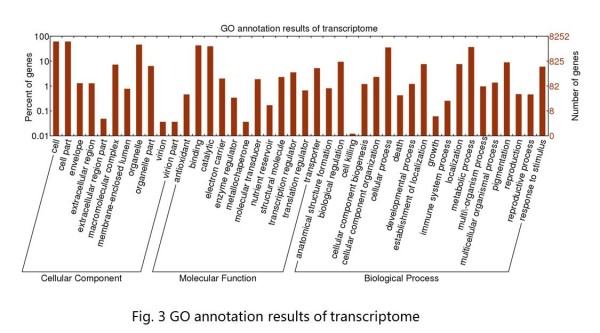
**Go annotation results of transcriptome**.

Of the 40,100 annotated unigenes, only 8,252 of the unigenes could be assigned at least one GO term, indicating that the peanut plant differs from model plants on genetic basis, and the search methodology of GO analysis is not suitable for peanut. These 8,252 unigenes were grouped into 42 GO functional categories (http://www.geneontology.org), which are distributed under the three main categories of Molecular Function (9,207), Biological Process (5,660) and Cellular Components (6,739) (Figure 3). Within the Molecular Function category, genes encoding binding proteins (43.45%) and proteins related to catalytic activity (39.88%) were the most enriched. Proteins related to metabolic processes (30.42%) and cellular processes (29.6%) were enriched in the Biological Process category. With regard to the Cellular Components category, the cell (33.02%) and cell part (33.01%) were the most highly represented categories. A total of 146 genes were annotated with the category of lipid biosynthetic process (GO: 0008610), and 69 genes were classified into the fatty acid biosynthetic process group (GO: 0006633) in the next level. Further analyses of these genes should provide information about fatty acid metabolism in peanuts.

A total of 18,028 unigenes were annotated with 125 pathways in the KEGG database (V56.0, Oct. 1, 2010); metabolic pathways were the most enriched (3,899), followed by plant-pathogen interaction pathways (1,290). Some pathways, such as the fatty acid metabolism pathway and fatty acid biosynthesis, the functions of which are clearly linked to the changes in the seed oil that take place during peanut ripening, would characterise in more detail in another paper (Zhang et al., unpublished).

### SSR mining from the peanut seed transcriptome

**Figure 4:**
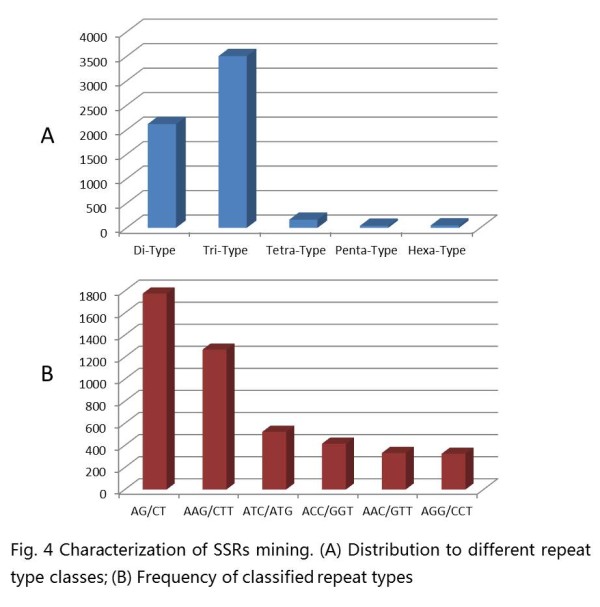
**Characterization of SSRs mining**. (**A**) Distribution to different repeat type classes; (**B**) Frequency of classified repeat types.

Microsatellite markers (SSR markers) are some of the most successful molecular markers in the construction of a peanut genetic map and in diversity analysis. In this study, 5,883 microsatellites were detected in 4,993 unigenes, of which, 728 sequences contained more than 1 SSR. The microsatellites included 2,120 (36.0%) dinucleotide motifs, 3,506 (59.6%) trinucleotide motifs, 166 (2.8%) tetranucleotide motifs, 42 (0.7%) pentanucleotide motifs and 49 (0.8%) hexanucleotide motifs (Figure 4A). The most abundant repeat type was (AG/CT), followed by (AAG/CTT), (ATC/ATG), (ACC/GGT), (AAC/GTT) and (AGG/CCT), respectively. (Figure 4B). Based on the 5,883 SSRs, 3,919 primer pairs were successfully designed using Primer3 (Additional file 2: Table S2). A total of 160 primer pairs (Additional file 3: Table S3) were randomly selected to validate these polymorphisms in six varieties. All 160 of the markers yielded amplification products, and 65 (40.63%) exhibited polymorphisms among the six varieties.

## Conclusion

In this study, we performed a global characterisation of the peanut transcriptome by RNA-seq using next-generation Illumina sequencing. We generated 26.1-27.2 million paired-end reads, comprising 59,077 unigenes from three different varieties of peanut with different oil contents. These unigenes were annotated with 42 GO functional categories and 125 pathways. A total of 5,883 microsatellites were identified among the 59,077 unigenes, and 3,919 primer pairs were developed based on the sequence library. These data will facilitate gene discovery and functional genomic studies in peanuts. We gained insight into the complex transcriptome of the peanut and established a biotechnological platform for future research.

## Authors' contributions

JZ carried out the peanut seed RNA isolation and sequence data analyses and drafted the manuscript. SL and JD designed the experiment and assisted in manuscript preparation. SC, JW, ZC, QZ, XL and YL prepared the plant materials and co-designed the experiments. All of the authors read and approved the final manuscript.

## Supplementary Material

Additional file 1 **Table S1**. The information on the redundancy of the data in this study with publicly available data. Click here for file

Additional file 2 **Table S2**. The primer pairs were successfully designed by Primer3 for all microsatellite. Click here for file

Additional file 3 **Table S3**. 160 random markers were used to validate the amplification and polymorphism. Click here for file
